# Proceedings of the Hahnemannian Association of Pennsylvania

**Published:** 1888-01

**Authors:** Wm. Jefferson Guernsey

**Affiliations:** Secretary


### PROCEEDINGS OF THE HAHNEMANNIAN ASSOCIATION OF PENNSYLVANIA.

A stated meeting of the "Hahnemannian Association of Pennsylvania," was held at the Continental Hotel, Tuesday, December 13th. Dr. Mahlon Preston, of Norristown, presiding. Present, all the active members, two honorary members, four associates, and two visitors.

Dr. Geo. H. Clark, of Germantown, read a paper on *Asterias rubens*, after which he was granted time to read a proving of *Dulcamara*. The prover, a lady, had been directed to take three doses, three hours apart. She took the first at 8 A. M., and at 8.20 noted some symptoms of the drug. Among others was the great feeling of "relaxation," which was very marked and which the Doctor said went to confirm other records. (Paper on *Asterias* is given in this issue.)

Discussion:

Dr. C. Carleton Smith—What temperament was prover?

Dr. Clark—Lymphatic.

Dr. John V. Allen—Did she know what she was taking?

Dr. Clark—No.

Dr. Robt. Farley—Your paper alluded to a pressure in the womb; what direction was that?

Dr. Clark—Downwards and forward.

Dr. Smith—What menstrual symptoms were noticed?

Dr. Clark—Delayed, and with colic which ceased as the flow appeared.

Dr. Preston—What proving have you consulted in comparison?

Dr. Clark—Those in Hering and Allen.

Dr. Smith—Symptoms usually go on with the menses for a time, but under *Dulc.* they do not. They improve.

Dr. Preston—I once made a cure of a hard tumor of the left breast when the nipple was retracted and the patient had just cause to fear cancer from the fact that a number of her family had died of it. I gave her *Asterias rubens*.

Dr. Clark—Were the axillary glands swollen ?

Dr. Preston—Yes, a little.

Dr. Smith—Had she any ovarian trouble ?

Dr. Preston—I don't know. As the case improved an ordinary abscess was set up in the breast which went on to suppuration, discharged and healed.

Dr. Farley—I never heard of a case of ordinary abscess having contraction of the nipple.

Dr. Smith—She either had or would have had some serious ovarian affection—it is a sure sign.

Dr. Edmund J. Lee read a letter from Dr. Tyrrell, of Toronto, Ontario, regarding Kali cyanatum, which he believed would be of great service in cancer, and presented a number of powders of the medicine for those desiring to make provings. They were distributed for that purpose. (The letter is given in this issue.) Discussion:

Dr. R. B. Johnstone—Three or four years ago I saw a woman with a fearful cancer of the breast, which had been diagnosed as carcinoma by several very prominent physicians. The nipple was retracted and there were three large lumps. Her breast was so large as to require support. The lumps looked as though there was something running under the skin, although there was no fluctuation, and appeared as if contused. In proving *Primos verticillatus* a man had the appearance in the arms on holding them up as though there was something running down under the skin. On this indication I gave her the 2c. potency, and her nipple came out and she broke out with a pustular eruption and improved nicely.

Dr. Guernsey—Where was the eruption ?

Dr. Johnstone—About the arms and hips.

The article on *The Organon* being called next, Dr. Johnstone stated that he had prepared it but forgot to bring it.

Original paper called, and Dr. Robt. Farley, of Phœnixville, read one entitled *Taking the Case* (which is given in this number). Discussion:

Dr. Walter M. James—I had a patient who had erysipelas of right side of face, with great swelling ; the eye seemed entirely obliterated. Had much trouble to find the remedy, but finally noticed that it was extending to the left side rapidly, and gave *Lycop.*, which helped very much in an hour's time. In seventy-two hours she was well. I gave two doses, an hour apart. You speak of making light of cases; I once had a patient who had been to a "water-cure" establishment for treatment and had been given up to die. She would not sit still five minutes, but constantly move about and one dose of *Arsen.* helped.

Dr. Preston—Does any one know of a remedy having the symptom of thinking they are pregnant?

Dr. Allen—*Thuja* has "old maids think they are pregnant."

I had a case of facial neuralgia in a dentist who had suffered severely from it for years, and who had submitted to removal of part of the nerve, and all kinds of drugging without effect. *Belladonna* helped his attacks, and sometime ago he sent in haste for me, and I sent a few powders of *Bell.*, until I could get there. He then wrote a note, asking if he could use the hypodermic syringe. I replied, of course, that he could not, but on getting to his house two hours afterward learned that his lips and mouth had been so excessively sore that he could not take the medicine in the usual way, and so had dissolved the *Bell.* in water, and after thoroughly cleansing the syringe, had taken a little hypodermically with *instant relief.*

Dr. Clark—Which side was the pain on?

Dr. Allen—Right side.

Dr. Clark—Water injected hypodermically has been known to act like Morphia.

Dr. Lawton—The remedy would act quicker in that way though.

Dr. Guernsey—My uncle used to say that a more powerful effect could be obtained from a medicine by taking a little of the solution on the tongue, forcibly inhaling through the mouth until the lungs were thoroughly inflated, and then exhaling through the nose, thus reaching the entire mucous tract.

Cases for advice being next in order, Dr. Farley stated the following symptoms: Gastric weakness. Sensation of an "all gone feeling," worse when tired. Better by belching. Worse after eleven A. M., and again at nine or ten P. M. Breakfast makes him feel good for nothing; better by lying down. Feels as if struck in stomach. Every three or four months has an attack of pain in intestines, which begins early in morning, waking from sleep causes great sweat and not relieved till after bowels move. Bowels usually costive, but with these attacks loose. Scaly eruption on scalp. After sitting in a room full of tobacco smoke has throbbing headache. He was directed to give *Sulphur*, high.

Dr. Guernsey then presented the resolution which had been

"tabled" at the last meeting, condemning vaccination with either human or bovine virus as dangerous and of questionable efficacy, and recommending the vaccination with tartarized Antimony, or the administration of *Malandrinum*. Passed.

Dr. Smith—I never vaccinate, it is an abomination; I use Malandrinum.

Dr. Johnstone stated that two of his children had had serious trouble from vaccination and died of diphtheria in a year.

Dr. Guernsey had had precisely same results with one of his.

Appointments for next meeting:

*Organon*, Dr. R. B. Johnstone, Germantown, Phila.

*Original paper*, Dr. C. H. Lawton, Wilmington, Del.

*Materia Medica*, Dr. Wm. H. A. Fitz, Philadelphia.

Three physicians were elected to associate membership. Adjourned.

WM. JEFFERSON GUERNSEY, *Secretary*.

